# The Poor-Law Infirmary

**Published:** 1907-10-05

**Authors:** 


### Poor-law Department.

### THE POOR-LAW INFIRMARY.

### SOME ESSENTIALS TO ECONOMICAL WORKING.

The Abolition of Allowances.

If the allowance system proved satisfactory from the point of view of the hard working men and women for whose benefit it was instituted, if the catering in Poor-law institutions succeeded in meeting all requirements, there would be no need for alteration. There is a higher consideration to be aimed at, after all, than expense, and to provide the staff with food, abundant in quantity and excellent in quality is the first object for the caterer to consider. In the course of his investigations, however, into the best methods of attaining these results, if he reaches the conclusion that a high standard can be attained simultaneously with a general reduction of expenditure, is it not wiser to give him a free hand, and remove barriers, which, though originally set up to limit the cost of the food, are now acting as impediments to retrenchment? Fifty years ago every one was allowanced. Every domestic servant in the smallest family expected her weekly half-pound of butter, her pound of sugar, her quarter-pound of tea. These things were measured out jealously into doubtful paper bags every week by the mistress of the house, and the residue was carefully locked up in receptacles which now only linger on in the form of elegant dining-room ornaments. Nobody in the present day weighs out bits of sugar or tea for their maids. It is only in Poor-law institutions that this long-discredited system survives. Long ago in the modern hospital, in the modern orphanage it has fallen into disuse. And the reason is not far to seek. It has been discovered that the ordinary person consumes when left alone to eat what he wants, far less than the boldest economist would dare to suggest as the proper mean for an allowance. Let us take the matter of bread. The ordinary allowance of bread is one pound a day. In no allowance table do the authorities venture to set it lower than this. But examine into facts. How much bread does an ordinary person consume when allowed to eat as much at every meal as he wants? The exact system of accounts of Guy's Hospital reveals that the total amount consumed by the hospital household in the year, excluding patients, is an average of 245 pounds, or exactly 11 oz. per day. Reckoning bread at its ordinary price of a penny a pound, there is here a leakage of over a farthing a day per head in an institution where the allowance system is in force, and a farthing a day means £38 a year for every 100 inmates. It may seem a small amount, but it is a good deal to throw away, surely, over the least expensive item in the diet table, and without anybody being the better for it, or, indeed, being at all conscious that the money is wasted. When the item of meat is considered the waste is more glaring, and even less justifiable. At the Fulham Infirmary the allowance is 7 lbs. of meat a week with $1\frac{1}{2}$ lb. of fish once a week in lieu of meat, and poultry or bacon once a month also in lieu of 1 lb. of meat. At the Wandsworth Infirmary the allowance is 7 lbs. of meat a week, 1 lb. of bacon, and 1 lb. of fish, the superior officers getting a fowl or duck weekly in addition. At the Kensington Infirmary the allowance is 7 lbs. of meat and 1 lb. of bacon, with 1 lb. of fish in lieu of 8 oz. of meat once a week. The superior officers get the preposterous allowance of $10\frac{1}{2}$ lbs. of meat a week, in addition to 8 oz. of fish twice a week, $1\frac{1}{2}$ lb. of bacon, and poultry once a week in lieu of $1\frac{1}{2}$ lb. of meat. There are several voluntary institutions in London in which the average quantities of meat consumed by members of the household are accurately noted. At St. Mary's Hospital the amount of meat consumed per head is nearly 5 lbs. per week. At the Great Northern Central Hospital the average is 5.6 lbs. At the Royal Free Hospital the average nurse consumes exactly 5 lbs. a week.

Now take it that the allowance of 7 lbs. a week is one pound more than is consumed under good domestic management in an institution where no allowance system is in force. There is a loss of at least sixpence a week per head, reckoning the price of meat at the comparatively low figure of sixpence per pound. Sixpence a week per head mounts up to £130 a year for every 100 members of the household. It will be argued that the allowance is merely the maximum quantity, and that the steward has orders not to issue more than is required. It is true some such regulation generally appears on paper in institutions where the allowance system flourishes. But it may readily be believed that the tendency is for the higher figure to be attained, and that the steward with the best intentions as regards economy is grievously hampered by the indisputable right of the staff to have their legal quantum of provisions. Should he succeed in lowering the average he is exposed to incessant complaint, and it is hardly possible that a sense of injury should not be generated.

This is, perhaps, not the worst consequence of a mischievous system. It is the influence of allowances in favour of waste which is most to be dreaded. Where very one is entitled to a little more than he needs, the amount left over is bound to be considerable, and there is no object in saving the broken victuals when each day brings with it its own weight of fresh provisions. Hence the common practice in Poor-law institutions of clearing the tables, and distributing the cold meat, etc., either to casual workers and hangers-on, or to the capacious pig-tubs. Just as the tendency is for the highest priced joints to be most in request under a contract where a separate price is given for "officers' joints," so in practice what is allowed will be, if not eaten, at any rate provided. The extra pound of meat a head muddled away among Poor-law officials every week, without anyone being the better for it, is answerable for a gross total of certainly not less than £10,000 a year to municipal London.

